# Governing Data and Artificial Intelligence for Health Care: Developing an International Understanding

**DOI:** 10.2196/31623

**Published:** 2022-01-31

**Authors:** Jessica Morley, Lisa Murphy, Abhishek Mishra, Indra Joshi, Kassandra Karpathakis

**Affiliations:** 1 Oxford Internet Institute University of Oxford Oxford United Kingdom; 2 NHSX London United Kingdom; 3 Uehiro Centre for Practical Ethics University of Oxford Oxford United Kingdom; 4 Harvard T.H. Chan School of Public Health Harvard University Boston, MA United States

**Keywords:** digital health, artificial intelligence, health policy

## Abstract

**Background:**

Although advanced analytical techniques falling under the umbrella heading of artificial intelligence (AI) may improve health care, the use of AI in health raises safety and ethical concerns. There are currently no internationally recognized governance mechanisms (policies, ethical standards, evaluation, and regulation) for developing and using AI technologies in health care. A lack of international consensus creates technical and social barriers to the use of health AI while potentially hampering market competition.

**Objective:**

The aim of this study is to review current health data and AI governance mechanisms being developed or used by Global Digital Health Partnership (GDHP) member countries that commissioned this research, identify commonalities and gaps in approaches, identify examples of best practices, and understand the rationale for policies.

**Methods:**

Data were collected through a scoping review of academic literature and a thematic analysis of policy documents published by selected GDHP member countries. The findings from this data collection and the literature were used to inform semistructured interviews with key senior policy makers from GDHP member countries exploring their countries’ experience of AI-driven technologies in health care and associated governance and inform a focus group with professionals working in international health and technology to discuss the themes and proposed policy recommendations. Policy recommendations were developed based on the aggregated research findings.

**Results:**

As this is an empirical research paper, we primarily focused on reporting the results of the interviews and the focus group. Semistructured interviews (n=10) and a focus group (n=6) revealed 4 core areas for international collaborations: leadership and oversight, a whole systems approach covering the entire AI pipeline from data collection to model deployment and use, standards and regulatory processes, and engagement with stakeholders and the public. There was a broad range of maturity in health AI activity among the participants, with varying data infrastructure, application of standards across the AI life cycle, and strategic approaches to both development and deployment. A demand for further consistency at the international level and policies was identified to support a robust innovation pipeline. In total, 13 policy recommendations were developed to support GDHP member countries in overcoming core AI governance barriers and establishing common ground for international collaboration.

**Conclusions:**

AI-driven technology research and development for health care outpaces the creation of supporting AI governance globally. International collaboration and coordination on AI governance for health care is needed to ensure coherent solutions and allow countries to support and benefit from each other’s work. International bodies and initiatives have a leading role to play in the international conversation, including the production of tools and sharing of practical approaches to the use of AI-driven technologies for health care.

## Introduction

### Background

The use of advanced analytics in health care may potentially unlock considerable benefits for patients, clinicians, and health and care services. Research shows that analytical techniques falling under the umbrella heading of artificial intelligence (AI) can recognize pathology in medical images [1-4], identify new medicines [5], and predict hospital readmissions [6]. However, the use of AI in health raises safety and ethical concerns that still need to be addressed by appropriate governance mechanisms (policies, ethical standards, evaluation, and regulation) [7]. Safety issues can arise following real-world implementation of AI systems into frontline health care because of their unpredictable performance in diverse settings, unknown human-computer interactions, lack of clarity around accountability and liability, and lack of education or preparedness among the health care workforce [8-10]. Ethical issues regarding AI systems go beyond issues of privacy. Ethical issues can be epistemic, normative, or related to traceability and affect individuals, relationships, groups, society, institutions, and the health care sector [11,12]. Ethical and safety concerns must be *proactively* taken into account for AI in health care to be helpful rather than harmful. Ultimately, one could argue that the responsibility for effective governance of AI technologies—and data, being the main *ingredient* for AI—across the health system and related sectors lies with governments. By doing so, governments help ensure that individuals, society, and health systems capitalize on the benefits of AI technologies while proactively preventing harm or misuse at all levels (the dual advantage of ethical AI) [13].

AI is often embedded in other digital technologies, products, or services (referred to in this paper as *AI-driven technologies*) when implemented in a health care system, for example, triaging chatbots such as *Babylon* or *Ada* [14]. These AI-driven technologies rely on large volumes of data for the purposes of training, testing, and validation. These data are collected, stored, and processed in a complex development pipeline [15]. Not all tasks in each stage of the development pipeline will be completed by the same organization or within the same national borders. Data increasingly flow across national borders, interacting with various technical infrastructures, actors, and data and technology standards [16]. Although some international data-sharing agreements exist, including biobanks and international consortia for medical imaging databases, most data used in developing AI technologies fall outside such agreements [9]. Different countries have different data protection laws [17], different understandings of socially acceptable uses of data, different values, and different ethical standards [18]. Unless monitored carefully, this patchwork of protections potentially enables companies to *ethics shop* or *ethics dump*, that is, to choose the most convenient and least restrictive location to conduct a specific task in the algorithm development pipeline [19]. For health care, where poorly designed algorithms may cause physical or psychological harm to patients, it is essential that these inconsistencies are addressed and, thus, the risks of ethics shopping and dumping are mitigated; otherwise, countries with weaker data or ethics protections (typically lower-income countries) could become *training and development* grounds for AI-driven technologies that are then deployed *for real* in countries with stricter protections (typically higher-income countries). This pattern has been previously seen in the development of medical and biomedical products in the past and is not only exploitative but also highly unethical and likely to result in significant breaches of human rights.

A degree of international variation in governance approaches to AI for health care is understandable because of national variation. Government investment in AI for health care is partly motivated by the desire to be a world leader in the field, and different governments interpret the implications for the regulatory environment differently [20]. For example, the United States believes that regulation stifles innovation and seeks minimal governance [21]. The United Kingdom believes more firmly in the power of proportionate regulation to facilitate innovation by providing structure and clarity and relies on firmer approaches to governance to maintain public trust in emerging technologies [22]. In addition, different governments have different underpinning social values. In the United Kingdom, for example, values related to individuals, such as empowerment and autonomy, are central, whereas, in China, collective values such as harmony and the collective good are more prominent [23]. Explicit recognition of these differences is important as AI-driven technologies are *sociotechnical systems* and thus often have values embedded in their design. If international variations in social values are not recognized upfront, this could result in harm to individuals and to health care systems if an AI-driven technology with mismatched values were to be exported from country A and deployed in country B. For example, although it might be socially acceptable in country A to use AI-driven technology to highlight how *you* compare health-wise to other people in your social network, this might be considered socially unacceptable—to the point of damaging public trust in the use of AI—in country B. As important as it is to acknowledge differences such as these that arise from a specific cause, it is also important to note that other differences are likely because of the sheer pace of development and lack of time to collaborate and coordinate across the complexities of international variation. Hence, the range of international governance approaches to AI limits this technology’s ability to deliver the full range of promised benefits.

A lack of international standardization of governance of AI for health care could create technical barriers to the adoption and realization of benefits from the perspective of interoperability and overarching accuracy. Without standardization of hardware, software, training data sets, and requirements for local adaptation, there is no guarantee that a model trained and designed in one country or setting will achieve the same level of accuracy (specificity and sensitivity) in another [9]. Furthermore, without standardization of medical device regulations, there is no guarantee that accuracy issues will be identified before deploying AI-driven technology. For example, in China, IBM Watson for Oncology was deployed, having been trained on data from a US hospital, leading to inaccurate and potentially unsafe clinical advice to patients in other contexts [24,25]. These ethical considerations and the ever-widening gap between expectation and reality do little to bolster AI-driven technology investment. This is important as the gap between expectation and reality is considered responsible for the AI winter of the 1970s and 1980s [26].

A lack of policy cooperation can also hamper market competition. Health care systems worldwide will derive optimal benefits from investments in analytics, including AI, if there is an open and competitive ecosystem of innovators building on previous initiatives. The lack of unified standards and the diverse regulatory requirements force companies to develop different AI and other emerging technologies for different markets. Lack of uniformity in these areas makes it harder for start-ups and small businesses to compete with the incumbents [27], which may lead to their acquisition by larger companies. Unless addressed, AI-driven technologies will likely fail because of provider monopolies and vendor lock-in, as has happened with other technology transformation programs [28].

The consequences of a disconnect in AI governance at the international level are serious. This is true of AI governance in general but especially true in the context of health care for 2 reasons. First, health care is a safety-critical area where poorly designed AI-driven tools can cause serious physical or psychological harm to health care practitioners and patients; therefore, all nations should aim for the same high standard of safety, efficacy, and ethics for health AI products, tools, and systems. Second, as the COVID-19 pandemic has demonstrated, globalization has made the health of local populations dependent on the overall health of the global population. In other words, as is often quoted by those rightly calling for international vaccine equity, “we’re not safe, until we’re all safe.” Consequently, calls from academia, policy makers, and industries for greater international policy cooperation are unsurprising. International coordination would help reduce gaps in guidance and regulation, make quality and safety standards visible and clear, and create an accessible common reference for developers and users [29]. As Feijóo et al [20] highlighted, international cooperation has improved welfare and avoided undesirable outcomes in other technology areas. Therefore, it is essential to forestall any ethical, cultural, economic, and political repercussions from increased AI use in health care.

The need for international cooperation in AI governance has led to international initiatives. For example, 2 United Nations agencies—the World Health Organization (WHO) and the International Telecommunication Union (ITU)—established a Focus Group on Artificial Intelligence for Health (FG-AI4H) in July 2018. This group, which is developing a benchmarking process for health AI models and a standardized evaluation framework, could be the hub for further international coordination, debate and consensus on common policies and standards, and knowledge sharing. Other international initiatives, such as the Global Partnership on AI (GPAI), founded in June 2020, are starting to understand and bridge the gap between AI research and implementation, including developing worked examples of AI use in areas such as the COVID-19 response that should inform public policy development.

Achieving broader international agreement on policies governing AI for health care is a complex undertaking. More work is needed to determine commonalities and differences between governance approaches to AI for health care, identify effective approaches, and share knowledge between countries. Building this evidence base will help policy makers, academia, and industry understand the context, expectations, and drivers of health AI development and implementation.

Importantly, there is no explicit agreement on which governance mechanisms, even once agreed worldwide, will ensure that emerging AI-driven technologies that act autonomously and continually learn and adapt are safe [30,31]. Designing governance for new technology is fraught with uncertainty, and any governance mechanisms will need regular review [32]. Therefore, developing internationally agreed policies will require greater flexibility than previously seen in other international policy contexts. All countries involved will need to work closely together, be open about the policies they are developing, and—when dissent arises—focus on building consensus.

### Objective

The barriers to achieving internationally accepted governance for AI in health care are significant. However, it is an important and exciting problem for policy makers. The opportunities and potential negative consequences are great, and the international community cannot afford to wait. For this reason, we set out to understand the current health AI governance mechanisms that the governments represented in the Global Digital Health Partnership (GDHP; a collaboration of 30 countries and territories and the WHO, with 31 members in total, which was formed to support the effective implementation of digital health) who commissioned this work are developing. We seek to identify commonalities and gaps in approaches, common challenges, and examples of best practices. The expected outcome is a set of policy recommendations serving as the foundation for internationally agreed AI governance mechanisms.

## Methods

### Overview

This research was commissioned by the GDHP, a collection of governments and territories, government agencies, and the WHO, which was formed in 2018 to support the effective implementation of digital health services. The Strategy and Policy team at the National Health Service (NHS) AI Laboratory in England, which is embedded inside the health service’s technology policy arm, *NHSX*, led the research with guidance from a researcher at the Oxford Internet Institute.

When designing a research program, the choice of methods depends on the nature of the research problem [33]. Typically, research focused on central government actions is answered with methods associated with policy analysis [34], particularly concerning effectiveness, efficiency, ethics, short- and long-term evaluation, and making recommendations [35]. Such methods derived from sociology, anthropology, economics, and organizational management include agent-based modeling, surveys, controlled comparisons, ethnography (eg, participant observation), and discourse analysis [36,37]. Although such applied policy research can provide rich insights, these are methods that are primarily designed to understand the impact of a policy decision that has already been made. Identifying how to implement AI in health care systems safely, effectively, and proethically requires *prospective* policy research that aims to determine what the policy should be [38]. Similarly, health service research, influenced by evidence-based medicine, relies heavily on methodologies using inferential statistics and randomized controlled trials [39]. However, the simplistic assumptions of these methods are criticized for not capturing the complex realities of the health care environment [39].

Instead, what is needed are methods aligned with the complexity theory, which are capable of dealing with individuals within social structures while acknowledging that feedback from individuals can have significant, unpredictable impacts on structuration processes [40], such as the implementation of AI-driven technologies in health care. What is needed is a theory-led [41] recursive approach that does not separate technology and context but analyzes technologies in use to build theory (as opposed to testing). Therefore, we used a mixed methods approach comprising 4 different stages:

1. A rapid scoping review of the academic literature and a systematic review of policy following the method by Gough and Tripney [42,43].
2. A thematic analysis of policy documents published by selected GDHP member countries; the findings from this analysis and the literature review were then used to inform and contextualize the semistructured interviews and the focus group.
3. Semistructured interviews with relevant policy makers from the included GDHP member countries, exploring selected individual GDHP member countries’ experience of developing and using AI-driven technologies in health care. The transcripts were analyzed using inductive coding.
4. A focus group with professionals working in international health and technology to discuss the themes and proposed policy recommendations from activities 1 to 3.

Both the interviews and the focus group were used, with the former providing a deep understanding of approaches taken by individual GDHP members and the latter providing the opportunity for representatives from member states to compare and contrast their approaches to data and AI governance for health care. This provided invaluable insight into the different priorities, principles, and values underpinning the different approaches of the different member states.

### Phase 1: Literature Review and Policy Analysis

Neither the literature review nor the policy analysis was designed to produce final outputs in and of itself. Instead, they were conducted in a pragmatic fashion, constrained by time to 2 days of searching and 3 days of reading by 2 separate researchers each (either LM and JM or AM and KK) to identify the key underresearched policy areas to be discussed in the interviews and later the focus group. The literature review was, therefore, a *scoping* review rather than a systematic review, intended to provide an overview of the nature and extent of existing research rather than a complete overview of the literature in this domain [43]. The limitations of this approach are noted in the *Conclusions* section. Systematic reviews of other areas of data and AI in health care policy do already exist [18,44].

For the scoping review, papers were identified using the search terms (1) *AI* and *policy* and (2) *AI* and *regulation* to search Scopus, PubMed, and Google Scholar. Papers that were published before 2015, not in English, case studies of specific algorithms or AI-driven technologies, evaluations of specific algorithms or AI-driven technologies, and AI methodology papers were excluded. Papers published between 2015 and 2020, written in English, focusing on the governance (ethics, policy, and regulation) of AI and data for health care from any of the countries in the GDHP were included. In total, the abstracts of 260 papers were reviewed, and of these 260 papers, 32 (12.3%) were included in the review.

The final 32 papers were read by 2 researchers who analyzed them using an interpretive approach. As such, the codes used to analyze the papers were not selected in advance but rather derived from key concepts in the literature [45].

For the policy analysis, relevant governance documents (ethics, policies, or regulation) from the following countries were searched: Australia, Canada, India, Japan, the Republic of Korea, Singapore, the United Kingdom, and the United States. These countries were a convenience sample, representing countries that had confirmed their participation in the semistructured interview and been nominated by the GDHP as one of the more active members in this space. The documents were identified through (1) a Google search using *[country] health AI,* *[country] health AI policy,* *[country] health AI regulation*, and *[country] covid artificial intelligence*; and (2) exploration of available documents from a country’s main health institutions, including health ministries, digital health agencies, medical device regulators, and other medical standard bodies.

As with the literature, documents were read by 2 researchers and analyzed using an interpretive approach to extract the key concepts.

Using the constant comparative method, the concepts extracted from both the literature and policy documents were combined into 6 key themes (codes) used to inform the development of the interview guide and, eventually, analyze the interviews themselves. Multimedia Appendices 1 and 2 contain the themes and interview guide.

### Phase 2: Interviews and Focus Group

A total of 16 GDHP member countries were approached for interviews, of which 10 (63%) were available. The final list of interviewees represented a convenience sample based on responses to a previous NHSX survey on AI use by GDHP member countries [46] while seeking equitable distribution across the globe. Interviews were conducted by 2 researchers, 1 principal and 1 supporting, with translation services provided upon request (used by Uruguay and the Republic of Korea). The following countries participated in the interviews: Australia, Canada, Hong Kong, Italy, the Kingdom of Saudi Arabia, the Netherlands, the Republic of Korea, Singapore, Uruguay, and Wales (see Multimedia Appendix 1 for the discussion guide). The interviews were recorded and transcribed by an independent contractor.

The interview transcripts were analyzed using the 6 key *codes* identified from the literature review and policy analysis. A total of 2 researchers analyzed each interview independently and compared their coding. Where opinions on how to theme a particular statement or quote differed between the 2 researchers, a discussion was had until an agreement could be reached. The final codes and themes for each interview—once an agreement was reached—were written up in a joint spreadsheet. All researchers then collectively reviewed the analysis of all the interviews and condensed the codes into 4 higher-level themes: leadership and oversight, an ecosystem approach, standards and regulatory processes, and engagement with stakeholders and the public. These themes were then used to guide the focus group discussions (more details on each are provided in the *Discussion* section).

A total of 10 participants with expertise in international health and technology organizations were invited to join the focus group. Some GDHP member countries unable to participate in the interviews were invited to attend the focus group to ensure representation from the GDHP membership. A total of 6 participants attended the focus group (with GDHP representation from Estonia, India, and Canada). The participants were split into 2 discussion groups, each facilitated by 2 researchers. The 4 themes mentioned earlier were used to guide the discussion. The participants were presented with statements describing each theme and the logic behind it, and they were then given question prompts around the theme to guide the discussion. As the focus group was conducted remotely, these prompts and statements were shown on the screen using Google Slides. The participants could then either respond verbally or use the web-based whiteboard *Jamboard* and write up their points using its post-it functionality. Where participants did not use these post-its themselves, the researchers noted their points for them. Using post-its enabled connections to be made in near real time, and the participants could give feedback if, for example, 1 of the researchers suggested that 2 separate points might be causally related.

In this way, the focus group was more idea-testing than idea-generating. The group dynamic was particularly important as it allowed for differences of opinion to be discussed openly and used in a generative fashion rather than being seen as an *issue that must be overcome*. It also, as mentioned earlier, allowed the researchers to develop an understanding of the *why* behind key differences in approaches.

Once the focus group had concluded, all research team members conducted a joint synthesis session. The key points under each theme from both groups were discussed and condensed into key summaries from which recommendations were extracted. These are discussed in the following section, which, for the purpose of focusing on empirical results from *primary research*, primarily focuses on the results from the interviews and focus group.

## Results and Discussion

### Overview

As outlined previously, the focus group and the semistructured interview findings revealed 4 core areas in which international collaboration would be beneficial: leadership and oversight, an ecosystem approach, standards and regulatory processes, and engagement with stakeholders and the public. Notably—as the interview guide indicates—we were anticipating the COVID-19 pandemic to have an impact on the development of AI and data policies in GDHP member states. However, this topic came up relatively infrequently, given the extent to which the pandemic has (necessarily) pulled focus over the past 2 years. Consequently, the following discussion does not make significant reference to the impact of the COVID-19 pandemic, although the potential impact in terms of prioritization for policy makers is noted in the *Conclusions* section. The white paper’s complete list of policy recommendations is shown in Multimedia Appendix 3 [47].

Policy recommendations or frameworks for using AI-driven technologies in health care need to cover the entire AI life cycle. The development of AI-driven technologies is an iterative process involving scoping, designing and building, and then deploying the AI-driven technology with continuous monitoring followed by improvement as required (as per the AI lifecycle diagram produced by the Information Commissioner’s Office [48]) The interviewees and focus group participants agreed on the need for an international body responsible for working with national representatives to build capability and ensure the implementation of recommended policies for each phase of the AI life cycle.

### Business and Use Case Development

Developers of AI-driven technologies (the supply) are usually not integrated into national health care systems and, even with demand signaling, may not know the areas of greatest need (the demand). Therefore, national governments and international consortia are responsible for clearly outlining the needs of the global, national, and local health care systems that could derive maximum benefits from AI-driven technologies. The participants in this study emphasized that the success of AI-driven technologies hinges on demonstrating their value, effectiveness, and safety in a clinical setting and across the broader health system. The interviewees advocated setting a vision for using AI-driven technologies in the health system at a national rather than state or provincial level, with opportunities for local interpretation and implementation. They stressed that high-level strategic vision should reflect areas within a country’s health system where AI-driven technologies could most benefit the population’s health. Similarly, Wirtz et al [31] noted that the best way to prevent market failure and harm to society when governing AI is to steer the market toward the greatest need to maximize efficiency. The participants in this study argued that such stewardship would bring a clear focus to the energies and funding for AI-driven technologies in a health system and help overcome barriers currently experienced by developers in translating AI research into practice.

However, the participants emphasized the importance of setting a strategic direction at the right level of abstraction [49]. International agreement on the strategic direction is indicated in some instances, such as during the COVID-19 pandemic; however, in other times, national- or local-level needs should be identified. Furthermore, even when the strategic direction is set nationally, the participants advocated for flexibility in the national vision to allow for regional interpretation and adaptation for accuracy and context-specific implementation.

One issue that arose in this study that could affect the support and resourcing of health AI development was the current lack of understanding regarding what AI is and its relevance to health care. Misconceptions included AI being autonomous (instead of existing as a decision-support system), its applicability only to medical imaging, and confusion regarding its data requirements. Misconceptions may derive from confusing and hyperbolic depictions of AI in the media [50]. Suggestions for overcoming these difficulties included shifting the focus from theoretical and exploratory conversations on AI for health care to tangible examples of AI-driven technologies already used in health systems. Use cases of AI-driven technology in health systems are most powerful when they satisfy otherwise unmet needs, improve user experience, and improve health outcomes. For example, the potential for AI uses in medical imaging to support the diagnosis of COVID-19 and assess its impact on people’s lungs illustrates the power of a needs-based approach with an actual use case. Several countries reported improved funding, access to and aggregation of health data, and political and public will for large-scale deployment of AI-driven technologies during the COVID-19 pandemic. This saw countries, including the United States, the United Kingdom, and Japan, set up national COVID-19 chest imaging databases specifically for AI development.

### Design Phase

As AI-driven technologies for health care can pose significant risks to patient safety, *hard* governance mechanisms, such as internationally accepted standards and regulations, are needed. The aspects of the AI life cycle that warrant more stringent control prompted lively discussions among the participants in this study. They agreed that new regulations should be limited and that new regulations should only be introduced if current medical device regulations are not fit for the purpose because of the unique features of AI-driven technologies.

A development stage approach to policy development should ensure that each component in developing AI-driven technologies within the AI life cycle receives equal consideration. For example, the start of the AI life cycle requires internationally agreed standards for access to aggregated data sets by researchers and developers. Standards could encompass secure trusted research environments and privacy-preserving techniques such as differential privacy [51]. Creating international standards for accreditation and access to research environments would improve cross-border access to health data without compromising data security. Ensuring health data are secure and deidentified creates possibilities for linkage with other data sets within the international community, for example, data sets on air quality, to provide insights into wider determinants of health. The other end of the pipeline requires policies for validation and evaluation services (including access to expertise), the provision of synthetic data sets, and the creation of test beds in various sites. These policies would advance research beyond the initial stages and help build health care providers’ trust in the accuracy of AI-driven technologies regardless of their origins. It would alleviate blind spots in AI governance. Taking inspiration from Crawford and Calo [52], AI governance requires a social systems approach, as each stage involves complex sociotechnical relationships that need careful consideration.

A key topic considered by the participants in this study was not *what* policies, standards, and regulations were required but *how* they should be developed. The participants stressed the need for transparency regarding the evidence and rationale for the approval of AI-driven technology or other emerging technology. Decisions in the approval process should be made public and disseminated to various stakeholders (including patients, the broader public, health care professionals, academics, industry representatives, and local government actors). The participants strongly favored active stakeholder involvement in the development of governance mechanisms. According to Kemper and Kolkman [53], meaningful transparency, which aids external critique and is not merely *ethics washing*, is crucial for maintaining stakeholders’ trust. Moreover, Aitken et al [54] demonstrated that genuine stakeholder involvement ensures that the opinions of patients and the public form part of the solution instead of creating an additional problem.

However, several participants reported struggling to achieve successful engagement activities. The engagement methods mentioned included formal consultations, research with specific groups, and direct product feedback. The participants felt that the heterogeneity of the population limited meaningful public engagement. The most vocal groups and the most digitally literate groups might monopolize consultations. Canada (a country that prioritizes public engagement) noted the following:

It’s a fairly small portion of the population that can meaningfully contribute to a conversation like that so, frankly, a lot of that engagement ends up being sort of the loudest voices or even the folks that are sort of regularly around the table.

Conversely, other GDHP countries expressed indifference or did not prioritize public engagement. This may reflect diverse cultural contexts.

### Training and Test Data Procurement

Training effective AI algorithms requires data of sufficient quality, adequate in size, and representative of the intended population. To aggregate data available nationally (or regionally if more appropriate), countries must first ensure appropriate legislative and policy frameworks for sharing and linking data across often disparate systems. An appropriately secure environment for data storage is required alongside agreed processes for data extraction from this environment and for analysis within it. There was broad awareness of needing to meet these data infrastructure requirements across the countries in this research, with varying levels of maturity found.

Hong Kong’s Data Collaboration Laboratory, operated by the Hospital Authority, provides an excellent example of an initiative that achieves access to high-quality data. Data collection began in the 1990s when Hong Kong first established infrastructure to create comprehensive (covering a large section of its population) and deep (covering patient history over decades) repositories of clinical information. The Health Authority Data Collaboration Laboratory (HADCL) provides the policies and infrastructure that enable access to the data for AI model training and development. The HADCL anonymizes and stores a large subset of the Hospital Authority data, including demographic, diagnostic, test, radiological, and other categories of clinical data. These data are stored at a physical location and are only accessible on-site. The on-site infrastructure includes a large data computational platform (with sufficient levels of compute) for state-of-the-art data storage, processing, access, governance, security, and operations. Researchers apply to access the data and computational power, and data-sharing agreements ensure that the HADCL has the rights of use if the AI models developed are clinically useful. Having HADCL rights of use ensures that a path to procurement and impact exists for the models. Previous models, such as an AI model scanning hip x-rays for fractures, are under consideration for wider clinical deployment.

Given the sensitive nature of health data [55], patients and the public are unlikely to trust AI-driven technologies without guaranteed data protection from end-to-end development pipelines. Safeguarding health data requires security and commercial protections to ensure that the fair value of data assets is realized. Safeguards are an essential aspect of public–private data partnerships, particularly those with large multinational companies. As citizens’ health data move across borders, coherent international plans for the protection and value return of data assets are crucial. These protections will help maintain national, public, and health care professional support for AI-driven technologies in health care. Other industries recognize the importance of trust in the security, provenance, and accuracy of a product. Many industries use transparent, standardized documents, called *declarations of conformity*, to describe the lineage of a product along with the safety and performance testing it has undergone [56]. Declarations of conformity do not yet exist for AI in health care products.

### Building

Participants from across the GDHP were frustrated by barriers to translating research into practice (ie, deploying AI models from a laboratory in clinical settings). The difficulties faced included lack of funding, lack of skills, and poorly defined processes and regulations. The few suggestions on overcoming these barriers focused on greater alignment between supply and demand for AI-driven technologies through oversight of the entire AI life cycle.

The English National COVID-19 Chest Imaging Database (NCCID) set up by NHSX illustrates an effective supply and demand connection. The NCCID is a centralized database containing x-ray, computed tomography, and magnetic resonance imaging images from patients in hospitals in the United Kingdom (COVID-19 positive and negative). The project’s aim was 3-fold: (1) to provide training data to researchers, start-ups, and commercial companies to develop AI models capable of recognizing COVID-19; (2) to test the models against another section of the database reserved exclusively for validation; and (3) to select and deploy the best-performing models in clinical settings to assist frontline clinicians’ response to the COVID-19 pandemic. The NCCID data are provided for free to developers to facilitate the deployment of AI models into practice. To ensure that this commercial arrangement benefits the NHS and the public, developers using the NCCID must provide their AI models for free to the NHS for its use during the pandemic. This approach should enable the market (the supply) to meet the pressing needs of the UK health system and its patients (the demand) while benefiting both parties (ie, the NHS and developers).

The NCCID is also an example of concurrent data policy and AI policy development. AI-driven technologies require access to data; therefore, streamlining policies from these 2 domains is essential to drive AI development and effective governance. However, many participants in this study raised concerns about the disconnect among the policy domains of data, AI, and less-complex digital health products (such as apps) apparent in their respective countries. The participants considered the disconnect among different but highly interrelated policy domains at the national and international levels as problematic. They proposed an ecosystem approach to policy development to ensure all policies relating to the entire AI life cycle were consistent and joined up.

The participants in this study were keen to build in-house technology workforces. However, they currently rely heavily on collaboration with private industry partners to deliver on the promises of AI-driven technologies, including progressing early-stage research into deployed products in clinical practice. Embedding technical skills within a health system was thought to offer 2 advantages. First, it would drive further innovation. Second, it would facilitate better integration of clinical expertise into digital health design, digital teams, and deployment processes.

In addition, international leadership could help alleviate government nervousness about public–private partnerships by supporting mechanisms for external scrutiny of private industry partners, standardizing terms for sharing and accessing patient data, and securing fair commercial terms between public and private partners. Ultimately, international policy collaboration was considered as a means of protecting the interests of public health systems faced with increasing involvement from private technology companies. Just as policy developments should consider all stages of the AI life cycle, so too must policy makers consider all potential actors.

### Testing and Validation

Larson et al [57] suggested that the existing European medical device law was deficient in 6 respects: (1) conflation of the diagnostic task with the diagnostic algorithm, (2) superficial treatment of the diagnostic task definition, (3) no mechanism to directly compare similar algorithms, (4) insufficient characterization of safety and performance elements, (5) lack of resources to assess performance at each installed site, and (6) inherent conflicts of interest. The interviews and the focus group in this study focused on points 3, 4, and 5. Regarding algorithms, the need for more flexible, appropriate, and adaptable mechanisms for proving the efficacy of AI-driven technologies in health care, other than randomized clinical trials, was flagged. Testing and validation should include mandating open reporting of results and algorithmic code for error checking and assessing clinical benefits and cost savings over the status quo rather than effectiveness alone. The participants felt that showing the value of AI-driven technologies in health care alongside conventional methods was key to garnering further support for their development and use. However, lack of skills, capabilities, and knowledge within local regulator workforces was considered a significant barrier to remediating current and future gaps in medical device regulation.

There was a lack of consensus in this study on the confines of AI regulation and where responsibility for governance lay as a result of overlaps in data use and health care products. National governments’ burdens could be reduced if the skill gap was filled or made superfluous through policy development at an international level. For example, national governments could assume responsibility for local adaptation of international frameworks. This would allow for counterchecks of products certified for use in comparator countries. Cohesion across international regulatory frameworks was considered a primary benefit of international policy collaboration. It could help redress the imbalance of regulatory experience and skills between nations and support low-resource or less digitally mature health systems in confidently and safely adopting AI technologies. Importantly, counterchecking standards must not be based on the lowest common denominator [27].

The United States and Japan have already embarked on updating their regulatory mechanisms to deal with the unique aspects of AI-driven technologies. Both countries are considering workflow changes for adaptive AI models. Unlike locked algorithms, adaptive algorithms can continuously learn and change their performance even after market rollout (eg, improving overall performance or adapting to new use conditions). AI-driven technologies can transform health care delivery as deployed models perform better over time and receive new information. However, existing regulation approaches are not optimal for regulating adaptive AI, as most performance changes require re-evaluating the entire AI model.

The US and Japanese approaches to workflow modification allow AI developers to articulate prospective future changes to an algorithm through a *predetermined change control plan*. A predetermined change control plan would include information about the types of intended modifications (eg, changes to the model’s performance, input data, and intended use) and their implementation. The regulator would evaluate the predetermined change control plan as part of the standard premarket evaluation of the AI-driven technology. Subsequent changes to the AI model postmarket deployment could be evaluated against the approved change control plan; hence, implementing preapproved modifications would be straightforward. The US Food and Drug Administration’s discussion paper on regulatory framework modification labels this strategy part of a Total Product Life Cycle regulatory approach. It is specifically designed for AI-driven technologies. The Total Product Life Cycle approach also evaluates the manufacturers of AI-driven technologies to ensure that they have established quality systems and abide by good machine learning practices governing data acquisition, model training, tuning, testing, and model transparency. The international community would do well to evaluate this approach’s effectiveness and test it on a larger scale.

### Deployment

Collaboration and multidisciplinary working by policy makers, technologists, health care professionals, and academics are needed to ensure appropriate expertise throughout the AI life cycle, especially during deployment of the technology into practice. Supporting research and implementation collaborations at a local level (eg, within a specific hospital or city) would create local showcase projects of AI research translated into practice. The design and execution of AI-driven technology trials require multidisciplinary approaches to assess clinical efficacy, comparative benefit and cost-effectiveness, and impact on clinical pathways and practice. Guidance on good trial design and reporting is now available with the AI-specific extensions to the CONSORT (Consolidated Standards of Reporting Trials)–AI and SPIRIT (Standard Protocol Items: Recommendations for Interventional Trials)–AI guidelines [58].

The lack of international coordination for the governance of AI in health care may limit its adoption because of issues of trust. There is consensus that trust is a core condition for successful innovation in digital health, including AI [59]. Clinicians are unlikely to trust the evidence of AI efficacy if they cannot scrutinize it and verify its origins. Clinicians will demand that AI for health care meet established standards of evidence and safety from familiar regulatory bodies.

Half of the GDHP member countries in this study highlighted apprehension among their clinical communities regarding AI-driven technologies in health care. The main reasons for clinicians’ apprehension were concerns about data quality and privacy, a poor understanding of AI, fear of redundancy if technology replaces health care professionals, and anticipated extra work if AI-driven technologies disrupt existing workflows. Therefore, GDHP members recommended that international collaborations develop a comprehensive AI syllabus for clinicians. An AI syllabus should include a definition of AI, its use in digital health technologies, current examples of AI-driven technologies in health care (including clinical and operational pathways), and why AI-driven technologies are used (including benefits to end users and health systems compared with conventional methods). They noted that an international review should consider ways to incorporate this education into medical training rather than relegating it to a continuing professional development topic.

### Monitoring

The oversight of and strategic vision for AI-driven technologies in health systems varied considerably among the GDHP member countries in this study. All countries reported having an organization or body responsible for digital health and, therefore, AI integration into digital health technologies. However, the organization of these bodies and their roles or responsibilities was inconsistent. The remits of these organizations variously included facilitating research, overseeing procurement, setting strategy, regulation, deployment of technologies, and a combination of these aspects. The use of statutory powers by countries varied from an advisory capacity to influencing legislation and standards. The variation in oversight mechanisms reflected significant differences in the stage of AI technological development; some countries in the early stages of developing AI-driven technologies in health did not share the imperative for strict oversight.

Improving national oversight procedures will support improved collective intelligence at an international level. Establishing such reporting and knowledge-sharing mechanisms would mean countries could access safety information about AI technologies that they are considering or have started using, bringing earlier identification of potential harms or risks.

### Conclusions

AI-driven technology research and development for health care outstrips available AI governance globally. International collaboration and coordination could facilitate comprehensive and coherent AI governance and enable countries to support and benefit from each other’s work. The discussed policy recommendations aim to reduce the major governance barriers to implementing safe, effective, and ethical AI-driven technologies across the AI life cycle. Testing and adopting these recommendations by GDHP member countries would help develop common ground and a core set of policy recommendations for endorsement by the GDHP and other international bodies.

Organizations and initiatives such as the FG-AI4H of the WHO/ITU, the GDHP, and the GPAI could lead international conversations and produce practical tools for implementing AI-driven technologies for health care, including across borders, and indeed have started to do so, as indicated by the recent publication of the guidance document *Ethics and Governance of Artificial Intelligence for Health* from the WHO [60]. However, there is still a long way to go, and there are many other opportunities to define accepted practices for evaluating the efficacy and safety of health AI (something that has been pursued by the G7 during the United Kingdom’s 2021 presidency), invest in and share educational materials (for the public and health care professionals), and create international benchmarking standards for AI models in set contexts (currently under consideration by the FG-AI4H of the WHO/ITU).

Convening these discussions and working groups at a practice level (ie, with people developing AI-driven technologies and those leading implementation in clinical pathways) is beneficial in bridging cultural and political divides. It focuses the conversation on shared technical challenges and successes of health AI and helps create a common ground and shared purpose, which is fundamental to international coherence. It is, of course, important to recognize that convening these types of discussions and encouraging GDHP member states to direct resources toward data and AI policy will be difficult in the wake of the COVID-19 pandemic, which will, undoubtedly, have left all with other pressing priorities.

It is also important to acknowledge the limitations of this particular research, as no research is without flaws. Specifically, although the literature review was used to inform the interview guides and contextualize the discussions and analysis, the pragmatic approach taken means that there were undoubtedly gaps in the authors’ knowledge at the time of designing the interviews and analyzing the results. Scoping reviews also typically lack rigor and do not involve a quality assessment. Therefore, there is a risk that the included papers were based purely on their *existence* rather than their quality, and this could have resulted in a skew in the topic selection for the interviews and focus group [43]. In addition, the convenience sampling method used to identify interview participants was sufficient for starting a conversation about this important topic but does mean that a relatively narrow range of opinions was gathered from GDHP member states, and there could be elements of bias in the findings as a result. These limitations and challenges in the wake of the pandemic mean that this paper and research should be viewed as an initial investigation—the starting point for further research rather than the conclusion.

The next steps will include conducting a more critical analysis of the emerging policies related to data and AI in health care from England and international comparators, analyzing how these policies compare to the *ideal* set out in the literature, and hosting further discussions with policy makers and subject matter experts as to how any gaps between reality and the ideal might be closed. Hopefully, through these conversations, the more strategic implications for global public health of investing in data and AI policy will become clear, providing a justification to GDHP members—and nonmember states—wishing to invest time and resources in these areas *even* in the wake of the COVID-19 pandemic.

## Appendix

Multimedia Appendix 1 Semistructured interview guide.

Multimedia Appendix 2 Framework for the thematic analysis of semistructured interviews and focus groups.

Multimedia Appendix 3 Policy recommendations from the white paper.

## Abbreviations

AI: artificial intelligence
CONSORT: Consolidated Standards of Reporting Trials
FG-AI4H: Focus Group on Artificial Intelligence for Health
GDHP: Global Digital Health Partnership
GPAI: Global Partnership on AI
HADCL: Health Authority Data Collaboration Laboratory
ITU: International Telecommunication Union
NCCID: National COVID-19 Chest Imaging Database
NHS: National Health Service
SPIRIT: Standard Protocol Items: Recommendations for Interventional Trials
WHO: World Health Organization
