# Supplementary material for: Governing Data and Artificial Intelligence for Health Care: Developing an International Understanding
Source: JMIR Form Res. 2022 Jan 31;6(1):e31623. doi: 10.2196/31623 (PMC8844981; doi:10.2196/31623)
Supplement: Multimedia Appendix 1 [file formative_v6i1e31623_app1.docx]

## **Supplement 1: Semi-Structured Interview Guide**

Interviews with GDHP member countries were semi-structured covering the use of AI-driven technologies in the country’s health system, how AI-driven technologies are developed and regulated, and how the COVID-19 pandemic has impacted the use of this type of technology.

As the interviews were semi-structured, below are the primary questions, which are not an exhaustive list of questions asked of interview participants.

| **Warm up and context setting** | |  | **Current use of AI in healthcare** | |
| --- | --- | --- | --- | --- |
| 1 | Tell me a bit more about yourself, your role and your involvement in digital healthcare in your country |  | 6 | Who oversees and/or regulates the use of AI in your healthcare system? |
| 2 | On a high level can you tell us how your country’s health system is organized? |  | 7 | How is AI policy created in your country? |
| 3 | How are digital health technologies used in your health system? |  | 8 | How are stakeholders engaged in the development of such policies? |
| 4 | How do you define AI? (with examples) |  | 9 | What are the barriers you face to creating national policies for AI? |
| 5 | Walk me through if/how AI is used in healthcare in your country. |  | 10 | Would you prefer to develop AI policy at a national level, or have an international standard that you can tailor to your own country’s context? |

| **Development of AI for healthcare** | |  | **Innovation during or related to the COVID-19 pandemic** | |
| --- | --- | --- | --- | --- |
| 11 | How has this landscape of AI use in your country’s healthcare system developed? |  | 15 | How have you used AI in your response to the COVID-19 pandemic? |
| 12 | What barriers, if any, do you face to deploying AI in healthcare?  What successes, if any, have you had when deploying AI in healthcare? |  | 16 | Tell me about how the COVID-19 impacted your policies around:   - Digital health - Data sharing - AI |
| 13 | How do you share data with:   - Technology companies? - Other nations? - Academia? |  | 17 | Have you participated in any multinational research studies or programmes related to AI in healthcare related to COVID-19? |
| 14 | How do you regulate data sharing?  How do you regulate AI? |  | 18 | How has COVID-19 changed the use of AI in your health system? |
|  |  |  | 19 | Is there anything else you think we should know about AI use in your healthcare system and/or during COVID-19 that you would like to share with us? |
