# Supplementary material for: Governing Data and Artificial Intelligence for Health Care: Developing an International Understanding
Source: JMIR Form Res. 2022 Jan 31;6(1):e31623. doi: 10.2196/31623 (PMC8844981; doi:10.2196/31623)
Supplement: Multimedia Appendix 2 [file formative_v6i1e31623_app2.docx]

## **Supplement 2: Framework for Thematic Analysis of Semi-Structured Interviews and Focus Groups**

Thematic analysis of interviews and the focus group followed the analysis framework below. The themes within the analysis framework were selected *a priori* following a rapid review of the relevant literature and selected policy papers from GDHP member countries.

| **Category** | **Objective** | **Themes/ Questions** |
| --- | --- | --- |
| Contextual | Identifying what is already in place:   - Use of AI including during the COVID-19 pandemic - Development of AI/ digital health policy/ regulation - Development of governance arrangements | - Theme 1: A, B, C, D  - Theme 2: A, C, D, E, F, G  - Theme 3: A, B, C, D, E, F  - Theme 4: A, B, C, D  - Theme 5: A, B, C |
| Diagnostic | Examining what exists: why has this been developed/ used, what has shaped this environment | - Theme 1: A  - Theme 3: A  - Theme 5: D  - Theme 6: A, B, C, D, E, F |
| Evaluative | Appraising the effectiveness of what exists, and what gaps are missing | - Theme 2: B, E, F  - Theme 5: D |
| Strategic | Identify new areas for policy intervention, and the levers for implementation  Identify practical recommendations based on challenges identified | - Theme 1: B, C, D  - Theme 2: E, F  - Theme 3: B, E, F  - Theme 4: B, D  - Theme 5: C |

| **Theme 1: Oversight of digital health** | | |
| --- | --- | --- |
| **#** | **Sub theme** | **Definition** |
| A | Structure of the health system | The way in which the health system is structured, accessed and financed, including devolution of decision making and management to a regional or local level |
| B | Organizations and bodies responsible for regulating and overseeing digital health | Current and proposed bodies that oversee the development and deployment of digital health, such as government agencies, regulatory bodies etc |
| C | Organizations and bodies with sole digital health delivery remit | Current and proposed bodies responsible for digital health innovation and use, such as health service bodies, government departments etc |
| D | Digital health strategies and policies in place and/or in development | Strategies and policies for the use of digital health technologies, including AI, at a national or regional level. This includes those relevant to funding, deployment and data governance and security. |

| **Theme 2: Use of AI across health systems** | | |
| --- | --- | --- |
| **#** | **Sub theme** | **Definition** |
| A | Domains of AI deployment | Uses of AI across the health system including disease and disability management, service delivery and operations, clinical trials and research. |
| B | Barriers to AI deployment | Major barriers to the development and deployment of AI in healthcare, across all categories (including, for instance, fragmented structure of the public health system, lack of regulatory clarity, translation from research to application, etc.), and what is being considered/ deployed in response. |
| C | Use of AI in COVID-19 response (nationally) | The use of AI in the national pandemic response, including for direct healthcare delivery, preventative measures and strategic planning. |
| D | Intercountry AI collaboration (pre, during and post COVID-19) | Any collaborations that have been carried out or are ongoing with other countries in relation to the development and/or use of AI for healthcare. This also includes research collaborations being undertaken as part of the COVID-19 pandemic response. |
| E | Implementation of AI: technical architecture | The current technical architecture for deploying AI, and that which has been identified as a need/ priority area for further implementation. |
| F | Implementation of AI: skills and education | The measures being taken (or being considered) to upskill the health workforce to enable AI utilization in healthcare delivery. This includes technical upskilling more generally for AI, but also AI upskilling specific to health, alongside upskilling and readiness of healthcare practitioners to utilize AI in their workflows. |
| G | Consideration of biomedical ethics | The ethical issues that have been considered, and the ethical standards/ frameworks that have been upheld, when developing and deploying AI in healthcare. |

| **Theme 3: Development of AI** | | |
| --- | --- | --- |
| **#** | **Sub theme** | **Definition** |
| A | Research | Main areas of relevant research expertise, which domains are funded and how, whether it’s mainly in academia or in private companies. This also includes pipelines to harness expertise and research for deployable AI. |
| B | Funding for AI development | Total amount of government funding for AI in health, how it has been apportioned across different AI focus areas (if available), what are the plans to top this up in the future. |
| C | Data access and sharing | The processes by which healthcare data can be accessed for research and innovation purposes and conditions under which data can be shared, including relevant legislation and policy instruments. This includes sharing across the health system, academia and commercial partners. |
| D | Consideration of data ethics | Ethical frameworks for the governance of health data that are considered and/or adhered to for the use of data in AI development. |
| E | Validation of AI (technical and clinical) | Validation standards and procedures for AI development in healthcare, including for software standards, data representativeness, accuracy, and clinical associations. Current and future efforts to trial/use synthetic data for validation medical AI models. |
| F | Evaluation of AI (technical and clinical) | Evaluation standards and procedures for AI deployment in healthcare, including system and human factors. This also includes existing and future approaches to post-market surveillance for performance, safety and clinical outcomes. |

| **Theme 4: Regulation of AI** | | |
| --- | --- | --- |
| **#** | **Sub theme** | **Definition** |
| A | Responsibility and oversight of AI regulation | The major bodies that have oversight over regulating the use of AI across the health system. |
| B | Regulatory process | The workflows currently in place to regulate AI in healthcare, and how that is to be updated in the future. Particular attention towards unique features of AI/Software As a Medical Device as opposed to conventional medical devices and the divergence this requires from conventional regulatory processes. |
| C | Incentives and sanctions | Levers to enforce regulation and examples of sanction actions which may be taken if a company/individual violates regulatory process. |
| D | Changes in regulatory process due to COVID-19 | Amendments to the regulatory processes for AI in health, including exemptions, that have been made in response to the COVID-19 pandemic. |

| **Theme 5: Standards and interoperability** | | |
| --- | --- | --- |
| **#** | **Sub theme** | **Definition** |
| A | National standards in use | National technical standards relevant to health technologies that have been developed for use. |
| B | International standards being use | International technical standards relevant to health technologies that are used; adhered to as a whole or tailored for the national context. |
| C | New standards/ changes to standards due to COVID-19 | New technical standards for health technologies introduced in response to COVID-19. |
| D | Desire for international versus national standards | Preference amongst member countries for adherence to national versus international technical standards for health technologies. |

| **Theme 6: Stakeholder and engagement** | | |
| --- | --- | --- |
| **#** | **Sub theme** | **Definition** |
| A | Civic involvement in developing policies and process | The inclusion of insights and perspectives from patients and the public in the development of health technology policies and standards. |
| B | Engagement with healthcare professionals on AI development and deployment | Current and planned engagement (through consultations, etc.) with healthcare professionals on how AI is being developed and deployed in care settings, and how these approaches should be updated. |
| C | Engagement with academic bodies on AI development and deployment | Extent of engagement and collaboration with research and academic actors (now and planned for the future) to encourage production and deployment of health AI products, or to help shape policy. |
| D | Collaboration with industry | Extent of engagement and collaboration with commercial actors (current and future) to encourage production and deployment of health AI products, or to help shape policy. |
| E | Trust in and acceptance of AI (health system) | Current perceptions and levels of trust that actors in the broader health system associate with the use/potential use of AI in care delivery. |
| F | Trust in and acceptance of AI (patients and public) | Current perceptions and levels of trust that patient and public populations associate with the use/potential use of AI in healthcare. |
