# Supplementary material for: Governing Data and Artificial Intelligence for Health Care: Developing an International Understanding
Source: JMIR Form Res. 2022 Jan 31;6(1):e31623. doi: 10.2196/31623 (PMC8844981; doi:10.2196/31623)
Supplement: Multimedia Appendix 3 [file formative_v6i1e31623_app3.docx]

## **Supplement 3: Policy Recommendations from the White Paper**

| **Area** | **Policy recommendation** |
| --- | --- |
| Leadership and oversight | - **Define the need:** Countries need to take a “needs-based” approach to setting the vision and direction for the use of AI-driven technologies within their health system. A country’s use of AI should be based on the problems and opportunities in the health system where AI-driven technologies could have the most impact to improve people’s health outcomes. - **End-to-end oversight:** Oversight of AI-driven technologies within a health system needs to cover the whole AI life cycle, alongside other supporting activities such as research, funding, and workforce development. - **Provide regulatory clarity:** Regulatory clarity is required both within and between countries to enable AI developers to understand and manage the risk of introducing AI-driven technologies into a health system. |
| Ecosystem | - **Aligning innovation with healthcare need:** Setting of research priorities for AI, and associated allocation of funding, should be based on the needs of patients and the health system. - **Access to quality data:** Countries should work to aggregate and link data from across their health and social care system, to create high quality repositories for analysis by accredited researchers, with provision of secure analytics environments and/or with appropriate mechanisms for data extraction in place. - **Deployment pipeline:** The translation of AI research into digital healthcare applications should be supported by a robust deployment pipeline. - **Working across sectors:** There should be exploration of public-private partnerships to address relevant skills and funding gaps that are preventing and stalling AI-driven technology development. |
| Standards and regulation | - **Clear and comprehensive AI standards:** There is a need for national standards to set minimum evidence and expectations for the entire AI life cycle, which should, where possible, be co-created with relevant disciplines and industries. - **International standards for benchmarking:** International standards should be developed to promote collaboration, with guidance for adaptation to national contexts and accounting for socioeconomic and cultural nuances. - **Robust regulation of AI throughout the life cycle:** Countries need to create robust regulatory processes that have a clearly defined scope and intention, recognizing the distinct nature of AI-driven technologies within regulatory models and delineating responsibility for each stage of the AI life cycle. These processes need to be transparent, proactive and flexible. |
| Engagement | - **Design with users:** The patient, HCPs and relevant stakeholders need to be involved in the design of AI-driven technologies from the start to ensure the resultant product or service meets clinical, user and professional needs and complements existing workflows and experiences. - **Demonstrable benefit:** Countries should focus on engaging and generating trust with the public, HCPs, industry, and other stakeholders through delivering AI-driven technologies that are concentrated on meeting a need(s) within the health system. Doing so moves the conversation about the public acceptability of AI away from the theoretical to one of showing the benefit and value AI-driven technologies bring to the health system. - **Invest in education:** Countries need to invest in wider public, professional and industry education on what is classed as AI, how AI-driven technologies are currently used in the health system and other industry, and what the benefit is to the end user especially compared to conventional methods. |
